# Superior vena cava drainage improves upper body oxygenation during veno-arterial extracorporeal membrane oxygenation in sheep

**DOI:** 10.1186/s13054-015-0791-2

**Published:** 2015-02-20

**Authors:** Xiaotong Hou, Xiaofang Yang, Zhongtao Du, Jialin Xing, Hui Li, Chunjing Jiang, Jinhong Wang, Zhichen Xing, Shuanglei Li, Xiaokui Li, Feng Yang, Hong Wang, Hui Zeng

**Affiliations:** Center for Cardiac Intensive Care, Beijing Anzhen Hospital, Capital Medical University, 2 Anzhen Road, Beijing, 100029 P.R. China; Beijing Institute of Heart Lung and Blood Vessel Diseases, 2 Anzhen Road, Beijing, 100029 P.R. China; Department of Cardiac Surgery, Beijing Anzhen Hospital, Capital Medical University, 2 Anzhen Road, Beijing, 100029 P.R. China; Department of Anaesthesia, Beijing New Century Women’s and Children’s Hospital, Wangjing North Road, Beijing, 100029 P.R. China; Institute of Infectious Diseases, Beijing Ditan Hospital, Capital Medical University, 8 East Jing Shun Road, Beijing, 100015 P.R. China; Beijing Key Laboratory of Emerging Infectious Diseases, Jingshundongjie 8, Beijing, 100015 P.R. China

## Abstract

**Introduction:**

Differential hypoxia is a pivotal problem in patients with femoral veno-arterial (VA) extracorporeal membrane oxygenation (ECMO) support. Despite recognition of differential hypoxia and attempts to deliver more oxygenated blood to the upper body, the mechanism of differential hypoxia as well as prevention strategies have not been well investigated.

**Methods:**

We used a sheep model of acute respiratory failure that was supported with femoral VA ECMO from the inferior vena cava to the femoral artery (IVC-FA), ECMO from the superior vena cava to the FA (SVC-FA), ECMO from the IVC to the carotid artery (IVC-CA) and ECMO with an additional return cannula to the internal jugular vein based on the femoral VA ECMO (FA-IJV). Angiography and blood gas analyses were performed.

**Results:**

With IVC-FA, blood oxygen saturation (SO_2_) of the IVC (83.6 ± 0.8%) was higher than that of the SVC (40.3 ± 1.0%). Oxygen-rich blood was drained back to the ECMO circuit and poorly oxygenated blood in the SVC entered the right atrium (RA). SVC-FA achieved oxygen-rich blood return from the IVC to the RA without shifting the arterial cannulation. Subsequently, SO_2_ of the SVC and the pulmonary artery increased (70.4 ± 1.0% and 73.4 ± 1.1%, respectively). Compared with IVC-FA, a lesser difference in venous oxygen return and attenuated differential hypoxia were observed with IVC-CA and FA-IJV.

**Conclusions:**

Differential venous oxygen return is a key factor in the etiology of differential hypoxia in VA ECMO. With knowledge of this mechanism, we can apply better cannula configurations in clinical practice.

**Electronic supplementary material:**

The online version of this article (doi:10.1186/s13054-015-0791-2) contains supplementary material, which is available to authorized users.

## Introduction

Acute respiratory distress syndrome (ARDS) is a severe lung disease with a high mortality rate [1-4]. Extracorporeal membrane oxygenation (ECMO) can provide gas exchange independently of mechanical ventilation, either as a rescue intervention or to minimize ventilator-induced lung injury [5-7]. Because of encouraging outcomes from the CESAR trial [5] and success with ECMO in patients with influenza A (H1N1) and ARDS [8-10], standard veno-venous ECMO has been proposed as the modality of choice for severe acute respiratory failure (ARF) without cardiac dysfunction [11,12]. When hemodynamic support is needed, such as in ARF patients with cardiac failure, veno-arterial ECMO (VA ECMO) has been considered a substitute for veno-venous ECMO to provide substantial hemodynamic and respiratory support [13,14]. However, when the heart recovers a certain extent of force capacities, ARDS patients with femoral VA ECMO display lower partial pressure of oxygen (PO_2_) in the upper body than in the lower body, which has been termed differential hypoxia [15,16]. Differential hypoxia might also occur in patients with VA ECMO support under certain conditions, for instance, hemodynamic instability with severe pulmonary hypertension, and respiratory dysfunction caused by pulmonary edema or infection following cardiogenic shock [17].

It has been reported that differential hypoxia could cause insufficient oxygen supply to the vital organs, such as the brain and heart [17,18]. To avoid the risk of differential hypoxia, a number of critical care professionals recommend that the clinical application of VA ECMO is best avoided in ARDS patients [19]. Meanwhile, some specialists are trying to solve the problem by elucidating the underlying mechanisms of differential hypoxia. Because hypoxemia occurs in the upper body, dual circulation has been proposed as the major reason for differential hypoxia in patients with femoral VA ECMO (from the inferior vena cava to the femoral artery, IVC-FA) [14,15]. According to this theory, in VA ECMO, oxygenated blood from the ECMO circuit enters the descending aorta to perfuse the lower body, whereas the blood flow of the upper body is from the left ventricle [14]. To deliver more oxygenated blood to the upper body, some clinicians have suggested: (1) to modify FA cannulation to the axillary artery or carotid artery cannulation (IVC-CA) [20,21]; or (2) to use veno-arterio-venous ECMO by adding an additional venous reinfusion cannula in the internal jugular vein to IVC-FA (FA-IJV) [17].

Interestingly, Kitamura and colleagues [15] reported that differential hypoxia could be ameliorated when IVC-FA was modified with superior vena cava (SVC) drainage (SVC-FA). Unlike IVC-CA and FA-IJV, which directly deliver oxygenated blood to the upper body, SVC-FA does not alter the blood supply to systemic circulation. We hypothesized that oxygen saturation in the SVC and IVC might be different during IVC-FA, which then contributes to differential hypoxia. In the present study, we provided evidence that differential venous oxygen return is an important modulator of differential hypoxia in VA ECMO. Moving forward, we can account for the differential venous oxygen return and apply a more appropriate cannula configuration in clinical practice.

## Methods

### Animals

Twenty adult male crossbred sheep (2 years old, weight 40 ± 5 kg) were provided by the animal centre of Beijing Anzhen Hospital, Capital Medical University. The protocol for animal care was approved by the Ethics Committee on Animal Experimentation of Beijing Anzhen Hospital, Capital Medical University.

### ECMO circuit

The ECMO system consisted of a Quadrox-D hollow-fiber oxygenator with BIOLINE coating, a Rotaflow centrifugal pump (Maquet, Rastatt, Germany) with heparin-coated circuit tubing, a Sechrist oxygen/air blender and a water heater/cooler (Sarns/3M Healthcare, Ann Arbor, MI, USA). Carmeda heparin-coated cannulas (Medtronic, Minneapolis, MN, USA) were used in all animals. Blood flow was monitored using a Doppler flow probe placed on the arterial side of the circuit (Transonic, Ithaca, NY, USA). An oximeter (Medtronic, Minneapolis, MN, USA) was used to monitor venous blood oxygen saturation and hematocrit.

### ARF model supported with femoral VA ECMO

**Figure 1:**
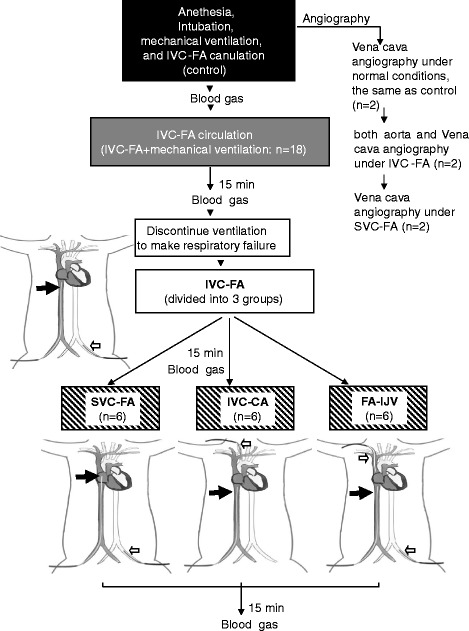
**Study protocol.** Heparin was infused to maintain an active clotting time of 180 to 220 sec after ECMO cannulation during the whole experiment. Of the 20 sheep, two were used for angiography. The other 18 sheep were randomly assigned to undertake one of three cannulation procedures. After 15 min of ECMO, ARF was initiated by removing the ventilator and discontinuing mechanical ventilation. The ARF animals were supported with IVC-FA for another 15 min and then were shifted to SVC-FA, IVC-CA or FA-IJV depending on the group assignment. The black arrow indicates the drainage cannula and the white arrow indicates the return cannula. Comparisons between IVC-FA and SVC-FA, IVC-FA and IVC-CA and IVC-FA and FA-IJV were made with paired *t* test. ARF: acute respiratory failure; ECMO: extracorporeal membrane oxygenation; FA-IJV: an additional return cannula was added into the internal jugular vein on the basis of femoral veno-arterial extracorporeal membrane oxygenation; IVC-CA: a drainage cannula was inserted into the inferior vena cava and a return cannula was inserted into the carotid artery; IVC-FA: a drainage cannula was placed into the inferior vena cava through the femoral vein and a return cannula was inserted into the femoral artery; SVC-FA: a drainage cannula was placed into the superior vena cava through the femoral vein and a return cannula was placed into the femoral artery.

The ECMO model was established as described previously with minor modifications [22-24]. Briefly, before anesthesia, all sheep were premedicated with dexmedetomidine (Dexdomitor; Orion Pharma, Madrid, Spain; 4 μg/kg) and morphine (Morfina 2%; B. Braun, Melsungen, Germany; 0.2 mg/kg) intravenously. Anesthesia was then induced with propofol (1% Propofol Lipuro; Fresenius Kabi AB, Beijing, China; 4 mg/kg) and maintained with sufentanil (5%; Yichang Humanwell Pharmaceutical Co., Ltd., Yichang, China; 5 μg/kg/h) and atracurium (0.2% Cisatracurium Besilate; Shanghai Hengrui Pharmaceutical Co. Ltd., Shanghai, China; 0.2 mg/kg/h) intravenously. After sheep were anesthetized, they were intubated with an endotracheal tube and connected to a mechanical ventilator (Servos-S, Maquet, Solna, Sweden) at a respiratory rate of 16 to 18 beats/min and a tidal volume of 6 to 8 ml/kg. An FA line was established to measure the arterial blood pressure (BP). A bolus of heparin (125 U/kg) was administered, and IVC-FA was established with 19-Fr and 15-Fr Carmeda heparin-coated cannulas (Medtronic) using the open Seldinger method. The venous cannula was placed within the IVC through the femoral vein. Placement of the cannulas was confirmed via ultrasonography. To stabilize the contribution of cardiac output (CO) and the pump flow to the body, we utilized dopamine, anesthesia and fluid to maintain heart rate (HR) and BP at a normal range. The pump flow was maintained at 50 ml/kg/min and 100% oxygen was administered at a flow rate equal to the blood flow rate. The sheep model of ARF was established as described previously through discontinuing ventilation (Figure 1) [25].

### Shifting femoral VA ECMO to other cannulation approaches

As shown in Figure 1, after 15 min of running, IVC-FA cannulation was shifted to (1) SVC-FA: the drainage cannula was moved to the SVC and the return cannula remained unchanged, (2) IVC-CA: the return cannula was moved to the CA with a 15-Fr Carmeda heparin-coated cannula (Medtronic) and (3) FA-IJV: an additional return cannula (12-Fr Carmeda heparin-coated cannula; Medtronic) was added in the IJV to IVC-FA. The total flow rate of ECMO was maintained at 50 mL/kg/min with 30% shunt flow.

### Blood gas analysis

Blood samples were collected by trocars at indicated time points and analyzed with a portable clinical blood gas analyzer (Abbott i-STAT, Chicago, IL, USA) (Figure 1). The sites where blood samples were obtained were as follows: (1) SVC: two centimeters distal to the orifice of the SVC; (2) pulmonary artery (PA): in the main pulmonary artery; (3) aorta: at the root of the ascending aorta (oxygen saturation (SO_2_) of the left atrium (LA) was also measured in IVC-CA); and (4) IVC: distal to the tip of ECMO drainage cannula.

### Angiography

Two sheep were used for SVC, IVC and aorta angiography (Figure 1) with a C-arm angiographic machine (Siemens, Munich, Germany). Contrast medium (Iodixanol; GE Healthcare, Pittsburgh, PA, USA) was injected using a high-pressure injector. For aorta angiography, the catheter was placed into the descending aorta near the return cannula in the FA. For vena cava angiography, the catheter was placed into the SVC or IVC.

### Statistical analysis

SO_2_ values are shown as the mean ± standard deviation (SD). The improvement of SO_2_ values based on different cannulation approaches was calculated and expressed as the difference of oxygen saturation values (ΔSO_2_). Statistical analysis was performed using SPSS 14.0 software (SPSS Inc., Chicago, IL, USA). The differences before and after discontinuing ventilation or before and after cannula shifting were analyzed with a paired *t* test. Differences among groups were analyzed with a Student’s *t* test or ANOVA. A *P* value less than 0.05 was considered statistically significant.

## Results

### Upper body hypoxia in the ARF sheep model supported with IVC-FA

**Figure 2:**
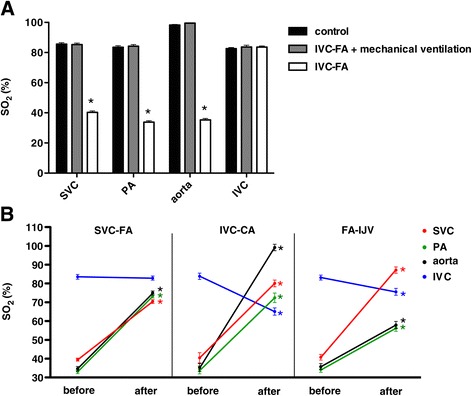
**SO**_**2**_ **in the ARF sheep model with different cannulations of VA ECMO. (A)** The cannulation of IVC-FA in normal sheep did not affect the SO_2_ of the SVC, PA, aorta and IVC. After establishing ARF in these sheep, the SO_2_ of the SVC, PA and aorta decreased; the SO_2_ of the IVC remained high. **(B)** The SO_2_ in the ARF sheep model with SVC-FA, IVC-CA, and FA-IJV. ‘before’ indicates the SO_2_ value of IVC-FA. ‘after’ indicates the SO_2_ value after cannulation shifting. ^*^Indicates *P* <0.01 between IVC-FA and mechanical ventilation or between IVC-FA and cannula-shifted sheep. ARF: acute respiratory failure; FA-IJV: an additional return cannula was added into the internal jugular vein on the basis of femoral veno-arterial extracorporeal membrane oxygenation; IVC-CA: a drainage cannula was inserted into the inferior vena cava and a return cannula was inserted into the carotid artery; IVC-FA: a drainage cannula was placed into the inferior vena cava through the femoral vein and a return cannula was inserted into the femoral artery; PA: pulmonary artery; SO_2_: oxygen saturation; SVC-FA: a drainage cannula was placed into the superior vena cava through the femoral vein and a return cannula was placed into the femoral artery; VA ECMO: veno-arterial extracorporeal membrane oxygenation.

Hemodynamic parameters, including HR and mean arterial pressure (MAP), were stable in each group of animals throughout the experiment and no significant differences were present among groups (see Additional files 1, 2, 3 and 4). Fifteen minutes after ARF, we observed that the SO_2_ of the SVC, PA and aorta were dramatically decreased (SVC: 85.3 ± 1.0% to 40.3 ± 1.0%, *P* <0.01; PA: 84.2 ± 1.1% to 33.9 ± 0.9%, *P* <0.01; aorta, 99.5 ± 0.2% to 35.3 ± 1.0%, *P* <0.01), whereas the SO_2_ of the IVC remained stable (83.7 ± 1.2% to 83.6 ± 0.8%, *P* = 0.83). Thus, similar to the clinical cases, upper body hypoxia occurred in the sheep model with ARF supported with IVC-FA (Figure 2A).

### Angiography in IVC-FA

**Figure 3:**
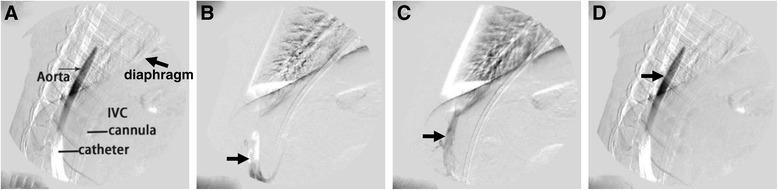
**Aorta angiography in IVC-FA. (a)** The diagram of aorta angiography. **(b)** Representative photos in the early stage of angiography. **(c)** Representative photos in the intermediate stage of angiography. **(d)** Representative photos in the late stage of angiography. The black arrow shows the contrast medium, which could only reach the diaphragm level. IVC-FA: inferior vena cava through the femoral vein and a return cannula was inserted into the femoral artery.

To investigate vessels to which the blood from the return cannula in the FA flowed, we performed aorta angiography. We observed that fully oxygenated blood from the return cannula could reach the diaphragm level, but could not supply the upper body under such conditions (Figure 3). An additional movie file shows this in more detail (see Additional file 5).

**Figure 4:**
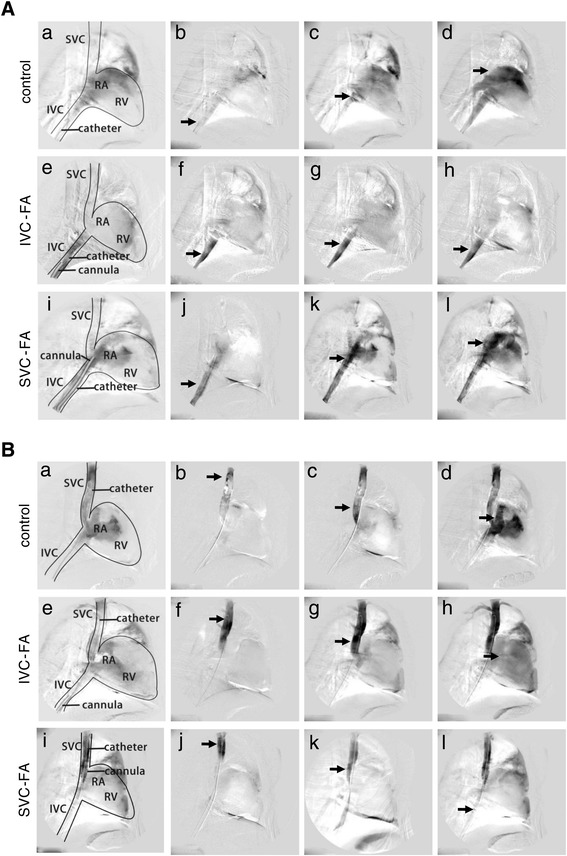
**Vena cava angiography in IVC-FA and SVC-FA.** The diagram (a, e and i) and representative photos in the early (b, f and j), intermediate (c, g and k) and late (d, h and l) stages of angiography are shown. **(A)** IVC angiography: contrast medium from the IVC. **(a-d)** IVC angiography in sheep without ECMO. **(e-h)** IVC angiography in sheep with IVC-FA. **(i-l)** IVC angiography in sheep with SVC-FA. Without ECMO, the contrast medium from the IVC entered the RA. In IVC-FA, the contrast medium from the IVC could not enter the RA. After shifting IVC-FA to SVC-FA, the contrast medium infused from the IVC could enter the RA again. **(B)** SVC angiography: contrast medium from the SVC. **(a-d)** SVC angiography in sheep without ECMO. **(e-h)** SVC angiography in sheep with IVC-FA. **(i-l)** SVC angiography in sheep with SVC-FA. Without ECMO or in IVC-FA, the contrast medium from the SVC entered the RA. After shifting IVC-FA to SVC-FA, the contrast medium infused from the SVC could barely enter the RA. The black arrow indicates the contrast medium. ECMO: extracorporeal membrane oxygenation; IVC-FA: a drainage cannula was placed into the inferior vena cava through the femoral vein and a return cannula was inserted into the femoral artery; RA: right atrium; RV: right ventricle; SVC-FA: a drainage cannula was placed into the superior vena cava through the femoral vein and a return cannula was placed into the femoral artery.

Vena cava angiography was performed in order to investigate whether the oxygenated blood entered the right atrium (RA). As shown in Figure 4 and the additional movie files (Additional files 6 and 7), under normal conditions without VA ECMO support, the contrast medium entered the RA from both the SVC and IVC. However, when the animals were supported with IVC-FA, the contrast medium failed to enter the RA from the IVC (Figure 4A; Additional file 8). In contrast, the SVC blood was conveyed to the RA (Figure 4B; Additional file 9). This observation indicates that high SO_2_ blood in the IVC was draining back into the drainage cannula instead of refluxing into the RA.

### SVC-FA improved upper body oxygenation

Next, we modified IVC-FA to SVC-FA by placing the tip of the drainage cannula into the SVC. Vena cava angiography revealed that the contrast medium entered the RA from the IVC but not from the SVC (Figure 4; Additional files 10 and 11). Furthermore, 15 min after shifting to SVC-FA, the SO_2_ of the SVC, PA and aorta significantly increased (SVC: 39.5 ± 0.6% to 70.4 ± 1.0%, *P* <0.01; PA: 33.2 ± 1.1% to 73.4 ± 1.1%, *P* <0.01; aorta: 34.7 ± 1.2% to 75.0 ± 1.1%, *P* <0.01), which indicated improved upper body oxygen supply. In addition, the SO_2_ value at the IVC remained unchanged (83.6 ± 1.3% to 82.9 ± 1.1%, *P* = 0.31) (Figure 2B).

### IVC-CA and FA-IJV improved upper body oxygenation

Next, we aimed to confirm the effects of IVC-CA on upper body oxygenation. The SO_2_ value of the aortic root was 99.2 ± 1.7%, whereas the SO_2_ value of the LA was 74.3 ± 2.3%. There was no significant SO_2_ difference between PA (72.4 ± 6.0%) and LA (74.3 ± 5.5%). The reason might be that oxygenated blood from the ECMO cannula in the CA caused a direct increase of the SO_2_ value at the aortic root. The SO_2_ value of SVC (80.1 ± 1.8%) and PA (72.4 ± 2.5%) were significantly higher than those in IVC-FA, which indicated that IVC-CA improved upper body oxygenation. Of note, the IVC SO_2_ value decreased under IVC-CA (83.9 ± 1.7% to 65.1 ± 1.9%, *P* <0.01) (Figure 2B).

Next, we achieved FA-IJV by shunting oxygenated blood from the returned cannula to the IJV. FA-IJV significantly increased the SO_2_ value at the SVC (40.8 ± 1.5% to 87.2 ± 1.7%, *P* <0.01) and improved upper body oxygen supply (PA: 34.3 ± 1.5% to 56.3 ± 1.7%, *P* <0.01; aorta: 35.8 ± 1.6% to 57.9 ± 1.9%, *P* <0.01). Of note, a decreased IVC SO_2_ value was observed in FA-IJV (83.2 ± 1.3% to 75.6 ± 1.8%, *P* <0.01) (Figure 2B). Moreover, as indicated by ΔSO_2_ values, the improvement in oxygen supply at the PA and the aorta was relatively lower in the animals supported with FA-IJV compared with those with SVC-FA and IVC-CA (*P* <0.01 for both). There was little change of ΔSO_2_ at IVC in SVC-FA sheep. In addition, there was a smaller decrease in SO_2_ at IVC in FA-IJV sheep than in IVC-CA sheep (Table 1).

**Table 1:** **The difference of oxygen saturation between IVC-FA and other approaches of cannulation**

	**SVC-FA**	**IVC-CA**	**FA-IJV**
SVC	30.9 ± 0.5	39.6 ± 1.7^*^	46.4 ± 1.4^*^
PA	40.2 ± 1.4	38.3 ± 1.2	22.0 ± 0.4^*#^
Aorta	40.3 ± 0.9	63.9 ± 1.3^*^	22.1 ± 0.6^*§^
IVC	−1.3 ± 0.9	−18.8 ± 1.8^*^	−7.6 ± 0.7^*#^

The SO_2_ values in IVC-FA were considered as basal level. The difference of oxygen saturation (ΔSO_2_) was obtained by subtracting the basal SO_2_ values from the SO_2_ values in SVC-FA, IVC-CA and FA-IJV, respectively. Basal levels were similar among sheep shifted to different cannulation. ^*^ *P* <0.01 vs. SVC-FA; ^#^ *P* <0.05 vs. IVC-CA; ^§^ *P* <0.01 vs. IVC-CA. FA-IJV: an additional return cannula was added into the internal jugular vein on the basis of femoral veno-arterial extracorporeal membrane oxygenation; IVC: inferior vena cava; IVC-CA: a drainage cannula was inserted into the inferior vena cava and a return cannula was inserted into the carotid artery; IVC-FA: a drainage cannula was placed into the inferior vena cava through the femoral vein and a return cannula was inserted into the femoral artery; PA: pulmonary artery; SO_2_: oxygen saturation; SVC: superior vena cava. SVC-FA: a drainage cannula was placed into the superior vena cava through the femoral vein and a return cannula was placed into the femoral artery.

## Discussion

Although VA ECMO plays an important role in treating severe respiratory and circulatory failure [11,26-29], unfortunately, the phenomenon of differential hypoxia limits its clinical application [17]. In the present study, we utilized a sheep model to mimic differential hypoxia in VA ECMO as determined by SO_2_ values. Importantly, we found a significant difference in SO_2_ values between the IVC and the SVC. These data indicate the existence of differential oxygen return in venous system. Through angiography, we demonstrated that better oxygenated blood was drained back to ECMO instead of returning to the heart. Based on this observation, we can explain how differential hypoxia is attenuated by alternative modes of cannulation.

**Figure 5:**
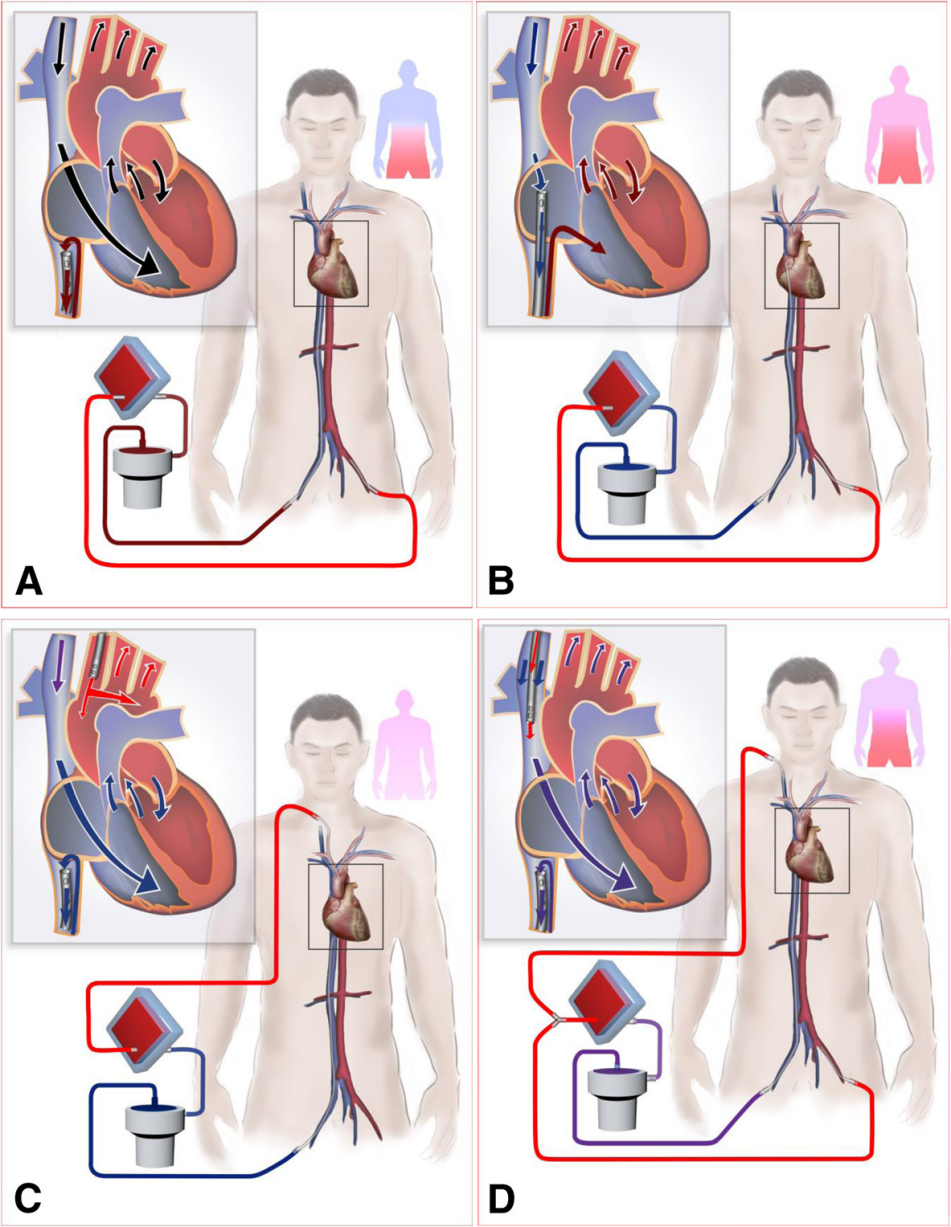
**Paradigm depicting the mechanism of differential oxygen return. (A**) Differential venous oxygen return between the IVC and the SVC exists in IVC-FA. Oxygen-rich blood is drained back to the ECMO circuit by the drainage cannula at the IVC, and the oxygen-poor blood from the SVC enters the heart and perfuses the upper body, which leads to differential hypoxia. **(B)** In SVC-FA, oxygen-poor blood in the SVC is drained to the ECMO circuit, whereas the oxygen-rich blood from the IVC enters the RA. **(C)** In IVC-CA, the oxygenated blood from the ECMO circuit is directly supplied to the whole body. **(D)** In FA-IJV, a certain amount of oxygenated blood is shunted into the SVC to improve upper body oxygenation. Differential venous oxygen return is attenuated in B, C, D. ECMO: extracorporeal membrane oxygenation; FA-IJV: an additional return cannula was added into the internal jugular vein on the basis of femoral veno-arterial extracorporeal membrane oxygenation; IVC-CA: a drainage cannula was inserted into the inferior vena cava and a return cannula was inserted into the carotid artery; IVC-FA: a drainage cannula was placed into the inferior vena cava through the femoral vein and a return cannula was inserted into the femoral artery; RA: right atrium; SO_2_: oxygen saturation; SVC-FA: a drainage cannula was placed into the superior vena cava through the femoral vein and a return cannula was placed into the femoral artery.

From angiography, we proved dual circulation by directly illustrating that blood flow to the lower body and the upper body was from the ECMO circuit and left ventricle, respectively. On one side, our results indicate that the femoral arterial reinfusion created a high level of lower body oxygenation when heart function is normal. This could lead to a high level of oxygen saturation in the returning venous blood from the lower body, as we saw from the high SO_2_ values in the IVC. This oxygen-rich blood was drained back into the ECMO circuit by the drainage cannula. On the other side, the oxygen-poor blood from the SVC entered the heart and perfused the upper body (Figure 5A). Thus, differential venous oxygen return between the IVC and the SVC was an important factor contributing to differential hypoxia. Draining the blood from the SVC where the returning blood is not as well saturated might be a key strategy for attenuating differential hypoxia (Figure 5B).

In IVC-CA (Figure 5C), through changing the arterial line of the VA ECMO circuit to the CA, the oxygenated blood from the ECMO circuit was directly supplied to the whole body. In addition, the oxygen supply to the heart increased indirectly because of the elevated SVC SO_2_ and/or LA SO_2_. Additionally, the venous oxygen return to the IVC was much lower than IVC oxygen of IVC-FA. In FA-IJV (Figure 5D), a certain amount of oxygenated blood, which originally perfused the lower body in IVC-FA, was shunted into the SVC to improve the upper body oxygenation. Additionally, the venous oxygen return at IVC (drainage) decreased, but not to the extent of SVC-FA and IVC-CA. As indicated by the ΔSO_2_, FA-IJV displayed lower efficacy in oxygenation improvement in cases where the pump flow remained at a constant rate compared with SVC-FA and IVC-CA. Although FA-IJV was less efficacious when compared with the other two cannulation approaches using the same pump flow, this deficiency can be abrogated in the clinic with additional flow to the SVC to meet the demands of the upper body.

Of note, we observed that SO_2_ in the aortic root was higher than in the PA in IVC-CA. However, because the PA and the LA displayed similar SO_2_ levels, potential confounding factors such as anesthesia levels could be excluded. An increase in aortic root SO_2_ might be a result from oxygenated blood from the ECMO cannula in the CA. This notion was supported by a recent finding [30] that determined that well-contrasted blood from the ECMO circuit (which was oxygenated) met low-contrasted blood from the left ventricle at the level of aortic root.

Although our present study explores how differential venous oxygen return results in differential hypoxia in IVC-FA, it is not limited to ECMO-supported patients. In minimal invasive cardiac surgery or in cardiac surgery requiring resternotomy (redo surgery), cardiopulmonary bypass is achieved through femoral cannulation. If the ventilation was stopped to facilitate operation before aortic cross-clamping or after declamping, differential hypoxia might occur when the heart ejects blood. This hypoxemia might lead to severe clinical consequences, such as brain death and ventricular fibrillation. Thus, ventilation should be maintained under such conditions.

### Limitation

Differential venous oxygen return is strongly dependent on CO. If CO is very low, differential hypoxia is less a problem. We did not measure CO in this experiment. However, the hemodynamic parameters including BP and HR maintained stable during this study. Measurements of CO will be included in future studies.

## Conclusions

In conclusion, our study indicates that differential venous oxygen return is a key factor in the etiology of differential hypoxia in VA ECMO. This observation provides new insight into the theory of dual circulation as well as clinical strategies of VA ECMO in pulmonary failure patients.

## Key messages

- Differential hypoxia is a pivotal problem in cardiopulmonary failure patients with veno-arterial extracorporeal membrane oxygenation (VA ECMO) support.
- Dual circulation has been proposed as the major reason for differential hypoxia in femoral VA ECMO (a drainage cannula is placed within the inferior vena cava through the femoral vein and a return cannula is in the femoral artery (IVC-FA)).
- The present study demonstrated that blood flow of lower body is from the ECMO circuit, whereas the blood flow of upper body is from the left ventricle.
- Differential venous oxygen return between IVC and superior vena cava under IVC-FA support is an important contributor to low oxygen saturation in the upper body.
- Draining the blood at the site where the returning blood was not as well saturated with oxygen may be a key strategy for attenuating differential hypoxia.

## Additional files

Additional file 1:
**Hemodynamic and blood gas changes from the baseline to IVC-FA.**
Additional file 2:
**Hemodynamic and blood gas parameters of SVC-FA.**
Additional file 3:
**Hemodynamic and blood gas parameters of IVC-CA.**
Additional file 4:
**Hemodynamic and blood gas parameters of FA-IJV.**
Additional file 5:
**Aorta angiography in IVC-FA.** The catheter for contrast medium injection was placed into the descending aorta near the return cannula in the femoral artery. The blood from the return cannula could reach the diaphragm level, but could not supply the upper body. IVC-FA: drainage cannula is placed within the inferior vena cava through the femoral vein and return cannula is in the femoral artery. Additional file 6:
**Vena cava angiography under normal conditions.** The contrast medium was from the inferior vena cava and entered the right atrium. Additional file 7:
**Vena cava angiography under normal conditions.** The contrast medium was from the superior vena cava and entered the right atrium. Additional file 8:
**Vena cava angiography in IVC-FA.** The contrast medium was from the inferior vena cava. When the animals were supported with IVC-FA, the contrast medium failed to enter the right atrium. IVC-FA: drainage cannula is in the inferior vena cava and return cannula is in the femoral artery. Additional file 9:
**Vena cava angiography in IVC-FA.** The contrast medium was from the superior vena cava. When the animals were supported with IVC-FA, the contrast medium entered the right atrium. IVC-FA: drainage cannula is in the inferior vena cava and return cannula is in the femoral artery. Additional file 10:
**Vena cava angiography in SVC-FA.** The contrast medium was from the inferior vena cava. When the animals were supported with SVC-FA, the contrast medium entered the right atrium. SVC-FA: drainage cannula is in the superior vena cava and return cannula is in the femoral artery. Additional file 11:
**Vena cava angiography in SVC-FA.** The contrast medium was from the superior vena cava. When the animals were supported with SVC-FA, the contrast medium failed to enter the right atrium. SVC-FA: drainage cannula is in the superior vena cava and return cannula is in the femoral artery.

## Abbreviations

ARDS: acute respiratory distress syndrome
ARF: acute respiratory failure
BP: blood pressure
CA: carotid artery
CO: cardiac output
ECMO: extracorporeal membrane oxygenation
FA: femoral artery
FA-IJV: an additional return cannula was added into the internal jugular vein on the basis of femoral veno-arterial extracorporeal membrane oxygenation
HR: heart rate
IJV: internal jugular vein
IVC: inferior vena cava
IVC-CA: drainage cannula is in the inferior vena cava and return cannula is in the carotid artery
IVC-FA: drainage cannula is placed within the inferior vena cava through the femoral vein and return cannula is in the femoral artery
LA: left atrium
MAP: mean arterial blood pressure
PA: pulmonary artery
PO_2_: partial pressure of oxygen
RA: right atrium
SO_2_: oxygen saturation
SVC: superior vena cava
SVC-FA: drainage cannula is placed within the superior vena cava through the femoral vein and return cannula is in the femoral artery
VA ECMO: veno-arterial extracorporeal membrane oxygenation
